# High influenza vaccination coverage among healthcare workers in acute care hospitals in Finland, seasons 2017/18, 2018/19 and 2019/20

**DOI:** 10.2807/1560-7917.ES.2022.27.17.2100411

**Published:** 2022-04-28

**Authors:** Charlotte C Hammer, Outi Lyytikäinen, Dinah Arifulla, Saija Toura, Hanna Nohynek

**Affiliations:** 1 Department of Health Security, Finnish Institute for Health and Welfare (THL), Helsinki, Finland; 2 European Programme for Intervention Epidemiology Training (EPIET), European Centre for Disease Prevention and Control (ECDC), Stockholm, Sweden

**Keywords:** influenza, vaccination, healthcare workers, Finland, vaccination coverage

## Abstract

**Background:**

Influenza vaccination is widely recommended for healthcare workers (HCWs) in European countries, but the coverage is not always satisfactory. In Finland, a new act was introduced in March 2017, according to which it is the employer’s responsibility to appoint only vaccinated HCWs for servicing vulnerable patients.

**Aim:**

We determined the influenza vaccination coverage among HCWs in Finnish acute care hospitals in three influenza seasons after introduction of the act.

**Methods:**

We analysed data collected by an internet-based survey sent annually to all Finnish acute care hospitals and described the influenza vaccination coverage among HCWs during seasons 2017/18, 2018/19 and 2019/20. We calculated mean coverage per healthcare district and season.

**Results:**

In season 2017/18, 38 of 39 hospitals, in 2018/19, 35 of 36 hospitals and in 2018/19 31 of 33 hospitals provided data. The mean influenza vaccination coverage was 83.7% (SD: 12.3) in season 2017/18, 90.8% (SD: 8.7) in 2018/19 and 87.6% (SD: 10.9) in season 2019/20. There was no significant increase or decrease in the mean coverage across the three seasons. The differences between districts were only significant in 2018/19 (p < 0.005).

**Conclusions:**

The coverage of influenza-vaccinated HCWs in Finnish hospitals was high in all three seasons and the current legal situation (semi-mandatory system) in Finland seems to provide a good background for this. Data collection should be maintained and improved for further monitoring.

## Introduction

Influenza can cause severe illness among high-risk groups such as elderly and immunocompromised people, leading to several million severe cases of illness and between 250,000 and 500,000 deaths annually [1]. Vaccination is a cornerstone in the prevention strategy for influenza. Higher influenza vaccination coverage among healthcare workers (HCWs) is associated with lower mortality of patients and also reduced staff sick days, leading to better care provision [2-5]. The European Council specifically referred to the importance of vaccinating HCWs in their recommendation on seasonal influenza vaccination in 2009 [6]. However, HCW vaccination coverage is often unsatisfactory [7,8]. Among the European Union countries between the seasons 2015/16 and 2017/18, only 12 countries provided data on HCW influenza vaccination coverage, with results ranging from 15.6% to 63.2% [9]. The barriers to vaccination among HCWs include lack of knowledge about both disease and vaccine, doubts about the safety and the effectiveness of the vaccine, dislike of injections, fear of adverse effects, inconvenience and limited support from managers [10,11]. Almost all European countries recommend influenza vaccination for HCWs, however, influenza vaccine uptake can be as low as 15% [9,12]. In Finland only one survey on this issue has previously been conducted in 2014, also showing a low coverage of 41% [13]. The influenza vaccination coverage varied widely between hospitals (18–86%) and notably nearly one third of the hospitals did not conduct surveillance on the coverage.

To improve immunisation uptake and protect patients, Finland introduced in 2017 the National Communicable Diseases Act [14], according to which it is the employer’s responsibility to appoint only vaccinated staff for servicing vulnerable populations – a so-called semi-mandatory approach. While the new vaccine decree came into force in March 2017, the relevant section – Section 48 (Box 1) – came into force 1 year later. We analysed data collected by an internet-based survey sent annually to all Finnish acute care hospitals and described the influenza vaccination coverage among HCWs during seasons 2017/18, 2018/19 and 2019/20. Our aim was to assess the influenza vaccination coverage among Finnish HCWs in the seasons following this new legal framework. Knowing that the data are collected locally with two different methods (with and without specific software), we were also interested as a side-inquiry if there was any difference between the coverage results of software users and those that do not use the software to ensure comparable data quality.

Box 1 **Communicable Diseases Act (1227/2016) Section 48: Vaccination of employees and students to protect patients, Finland**

1. For work in the client and patient facilities of social welfare and healthcare units which are used for treating clients or patients who, based on medical assessment, are susceptible to severe consequences from communicable diseases, a person with inadequate protection from vaccination may only be deployed in exceptional circumstances.
2. Employees and students in practical training must be protected against measles and varicella, either through vaccination or by having had the disease. In addition, vaccination against influenza is required, as is vaccination against whooping cough for persons treating infants.
3. Student healthcare services must ensure that students participating in practical training have the protection from vaccination referred to in subsection 2.
4. An employer has the right to process data of an employee or a student in practical training concerning the suitability for tasks referred to in subsection 1, as regards adequate protection from vaccination, with the consent of the employee or student and in accordance with the Act on the Protection of Privacy in Working Life (759/2004), the Occupational Health Care Act, and the Personal Data Act.

Source: [14].

## Methods

### Study context

The healthcare system in Finland (population 5.5 million) is organised in 20 geographically and administratively defined healthcare districts (HDs), with populations ranging from 63,000 to 1.8 million. Five HDs have a tertiary care hospital and 15 HDs have secondary care hospitals; the number of primary care hospitals vary annually and between HDs. Infection control teams based in the tertiary and secondary care hospitals have a consulting role in the infection control activities in their area; the responsibility for action is on local level.

### Data collection

Since the beginning of 2018, an internet-based survey requesting data on structure and process indicators related to infection control has been sent annually to all Finnish acute care hospitals. Psychiatric and long-term care facilities are excluded from the survey, as is one hospital on the Åland Islands. One of the survey questions concerns influenza vaccination coverage among HCWs, including nurses, doctors and other workers who have direct contact with patients. Specifications for the nominator and denominator to calculate the coverage are provided (Box 2) [15]. The survey is sent to staff in charge of infection control activities at all acute care hospitals providing tertiary, secondary and primary care. Knowing that many hospitals were increasingly using a software (ePiikki) for data collection, we did an additional survey by email to collect information about its usage in October 2020.

Box 2 **Specifications for calculating and reporting influenza vaccination coverage, Finland, 2018−2020**

- The numerator is the total number of vaccinated HCWs in special healthcare units on 31 December. HCWs include nurses and physicians, but not administrative and technical staff.
- The denominator is the total number of HCWs in special healthcare units at a given time, for example on Monday of week 44. Offsets are limited to those who were absent for more than 30 days on that day, regardless of the reason. HCWs includes nurses and physicians, but not administrative and technical staff.
- Depending on the framework, psychiatry may be considered, for example if it is located in the same building as the somatic specialist wards. Laboratory staff will be taken into account if their data are readily available. Most importantly, the numerator and denominator data in the formula should cover personnel in the same units/specialties.

HCW: healthcare worker.

### Analysis

The unit of analysis in the study was hospitals, as only aggregate data (vaccination coverage per hospital) were available. We performed a descriptive analysis of the vaccination coverage for the three seasons and across HDs. We also analysed differences between hospitals that used the ePiikki software and those that did not. For comparisons between HDs and between hospital types, we performed analysis of variance and for comparisons between seasons and between hospitals that used or did not use the software, we performed Walsh two-sided t-tests. Significance was assumed at p < 0.05. Analysis was performed in R (version 3.6.1 using RStudio).

## Results

In total, 39 hospitals provided data on influenza vaccination coverage among HCWs for at least one season and 31 provided data for all three seasons. In the 2017/18 influenza season 38 hospitals (survey sent to 39), in the 2018/19 season 35 hospitals (survey sent to 36) and in the 2019/20 season 31 hospitals (survey sent to 33 hospitals).

The mean influenza vaccination coverage was 83.7% (standard deviation (SD): 12.3; range: 65.5–95.0) in season 2017/18, 90.8% (SD: 8.7; range: 57.0–98.0) in season 2018/19 and 87.6% (SD: 10.9; range: 72.0–99.3) in season 2019/20 (Table 1). The differences between HDs were only significant in the 2018/19 season. No increase or decrease of the mean coverage in Finland was significant. The coverage across districts ranged from 65.5% to 95.0% in the 2017/18 season, from 57.0% to 98.0% in the 2018/19 season, and from 72.0% to 99.3% in the 2019/20 season (Table 2). The differences in annual changes between districts were not significant.

**Table 1:** Influenza vaccination coverage among healthcare workers in acute care hospitals by season and change between seasons, Finland, 2017–20

Season	Mean %	SD	Median %	IQR	p value (between districts)	p value (between hospital types)	p value (all of Finland, all hospital types)
2017/18	83.7	12.3	84	17	0.89	0.29	N/A
2018/19	90.8	8.7	91	9.5	**0.01**	**0.001**	N/A
2019/20	87.6	10.9	90	15.5	0.08	0.58	N/A
Change between 2017/18 and 2018/19	6.6	11.6	3	12.5	0.95	**0.02**	0.17
Change between 2018/19 and 2019/20	−2.9	9.4	0	5	0.11	0.31	0.27
Change between 2017/18 and 2019/20	4.3	11.2	2	10.5	0.80	0.21	0.80

IQR: interquartile range; N/A: not applicable; SD: standard deviation.

The Table shows comparisons between districts, hospital types and seasons for individual seasons and changes between seasons across all of Finland. Statistical significance indicated in bold.

**Table 2:** Mean influenza vaccination coverage among healthcare workers in acute care hospitals, by district, Finland, 2017–20

District	Mean % 2017/18	Mean % 2018/19	Mean % 2019/2	Mean change 2017/18–2018/19	Mean change 2018/19–2019/20	Mean change overall 2017/18 vs 2019/20
1	87.0	86.0	86.0	−1.0	0.0	−1.0
2	88.0	90.0	90.0	2.0	0.0	2.0
3	68.0	57.0	76.0	−11.0	19.0	8.0
4	85.6 (12.8)	98.0 (4.4)	99.3 (1.9)	13.6 (13.6)	−0.7 (1.9)	13.1 (14.3)
5	84.0	82.0	90.0	−2.0	8.0	6.0
6	81.0	86.0	81.0	5.0	−5.0 (21.9)	0.0
7	73.0 (35.4)	89.5 (4.9)	73.0 (26.9)	16.5 (30.4)	−16.5	0.0 (8.5)
8	83.0	88.0	86.0	5.0	−2.0	3.0
9	88.0	87.0	98.0	−1.0	11.0	10.0
10	77.0	No data available		N/A	N/A	N/A
11	95.0	95.0	73.0	0.0	−22.0	−22.0
12	88.0	92.0	83.0	4.0	−9.0	−5.0
13	85.0	90.0	92.0	5.0	2.0	7.0
14	87.0 (14.2)	91.3 (6.1)	88.3 (2.3)	4.3 (13.1)	−3.0 (6.9)	1.3 (16.4)
15	89.0	88.0	85.0	−1.0	−3.0	−4.0
16	78.3 (6.4)	89.0 (5.6)	75.7 (2.5)	10.7 (8.5)	−13.3 (5.5)	−2.7 (4.0)
17	92.0 (12.1)	93.7 (5.7)	92.0 (8.5)	1.7 (9.0)	−2.5 (0.7)	3.5 (6.4)
18	80.0 (1.4)	91.0	91.0	12.0	0.0	12.0
19	65.5(10.6)	72.0	72.0	14.0	0.0	14.0
20	95.0 (7.1)	95.0 (0.0)	97.0	0.0 (7.1)	2.0	7.0

N/A: not applicable.

Mean changes are subject to differing numbers of hospitals participating in each season per district. Standard deviations are shown in brackets only where reports from more than a single hospital were available.

The coverage by hospital type (primary, secondary, tertiary) ranged from 81.4 (primary care in 2017/18) to 94.6 (primary care in 2018/19) (Table 3). This is reflected in the significant change between seasons 2017/18 to 2018/19 (Table 1). Accordingly, the differences between hospitals types were significant after primary care hospitals had increased coverage in the season 2018/19 (Table 1). The coverage remained stable but lowest in secondary care hospitals across the seasons (mean across seasons: 84.7%). In tertiary care hospitals coverage also remained stable (mean: 91.2%). In primary care, coverage increased significantly from 81.4% in 2017/18 to 89.3% in 2019/20 (p = 0.021).

**Table 3:** Mean influenza vaccination coverage among healthcare workers in acute care hospitals, by hospital type, Finland, 2017–20

Hospital type	2017/18		2018/19		2019/20		Change 2017/18–2018/19		Change 2018/19–2019/20		Change overall 2017/18 vs 2019/20	
	Mean %	SD	Mean %	SD	Mean %	SD	Mean %	SD	Mean %	SD	Mean %	SD
Primary	81.4	14.5	94.6	6.3	89.3	15.4	13.3	14.3	−6.0	10.0	9.1	12.8
Secondary	83.2	10.0	85.5	9.9	85.4	7.7	1.9	6.4	−1.7	9.4	1.7	9.3
Tertiary	90.1	10.2	93.6	4.8	90.0	7.9	3.4	9.3	−0.1	7.7	1.3	11.1

SD: standard deviation.

Mean changes are subject to differing numbers of hospitals participating in each season per hospital type.

For the influenza season 2019/20, we assessed the impact of the use of the ePiikki software on reported influenza vaccination coverage. Of the 31 hospitals that provided data on vaccination coverage, 20 used the ePiikki software and 11 did not. The hospitals that used the ePiikki software reported a mean coverage of 88.3% (SD: 9.2) and hospitals that did not use the software reported a mean coverage of 86.5% (SD: 14.0). This difference was not statistically significant (p = 0.70).

## Discussion

The response rate was high throughout the seasons with annually only single hospitals not providing data. The percentage of vaccinated HCWs in Finland was high with a mean of 87.4% across the three seasons with no significant changes during the 3 years of data collection. There was a considerable range of mean coverages in different HDs but differences between the HDs were not significant either, except for the season 2018/19, which also saw the largest range with means across HDs from 57.0% to 98.0%. We are not aware of any immediate reasons for this unexpected result in 2018/19. 

While the coverage remained stably high in tertiary care hospitals across the three seasons and stable but lower in secondary care hospitals, coverage increased significantly in primary care hospitals. The increase in coverage in primary care hospitals was primarily driven by a sudden jump in coverage from season 2017/18 to 2018/19 which may have been a response to the change in the decree. However, it is unclear why we did not observe this effect in the other two hospital types as strongly, particularly in secondary care hospitals which had a comparably low if slightly higher coverage prior to the decree.

In comparison with other countries, the influenza vaccination coverage in Finland is high. English data from the seasons 2016/17 to 2018/19 showed coverages between 63% and 70% [16,17]. The highest reported coverages that we found were from the United States (US), ranging from 77% to 81% in the seasons 2014/15 to 2018/19 [18].

Notably, the methods used for data collection differ between countries. The current Finnish method is highly decentralised, with calculations of the percentage performed in each hospital and only percentage data reported to the Finnish Institute for Health and Welfare since the national register data cannot be used for this purpose. The Finnish vaccine register does not contain data on occupation nor information on whether the current work tasks require influenza vaccine, and the national register of healthcare professionals only includes data on current professional practice rights [19]. The system in England relies on Trusts, which are similar to HDs in Finland for data collection, however, the percentages are calculated centrally, with each Trust reporting cumulative numbers of influenza vaccine doses given to HCWs [20,21]. In addition, data in England are collected in five monthly surveys rather than once a year [20,21]. This makes it possible to analyse uptake throughout the influenza season, which the Finnish system does not currently allow. On the other end of the methodological spectrum, the US system uses self-reported vaccination uptake through an online panel [18,22]. The reliability of this approach is less clear.

Our study is subject to a number of potential limitations. The first is the availability of percentages of vaccinated staff only. As hospitals calculate the vaccination coverage among their HCW themselves, there is a higher likelihood of differences in calculation across hospitals/HDs. While there is an official suggested formula for hospitals to use, there is no way to ensure that this was done correctly. There is a risk that hospitals and HDs use different data and measures for their calculation of vaccination coverage. 

## Conclusion and recommendations

Despite the unclear data quality at the moment, a first important recommendation in the Finnish context but also more generally is to collect data on HCW vaccination coverage and to publish such data. However, the quality of the data should if possible be improved. HDs reporting crude data would be more advisable so that percentages can be calculated centrally with an ensured standardised methodology, allowing also to calculate the confidence intervals. In addition, it should be further investigated if weighting to the distribution of the population of healthcare personnel by occupation, age, sex, other demographic factors, work setting and region might yield additional useful information. It may be most suitable to do this in the form of an initial pilot study in two or three hospitals.

The coverage in Finland has been comparably high in the three seasons covered by our analysis. The current legal situation in Finland seems to provide a good background for this. Compared with the coverage survey conducted before the change of the law, the coverage was consistently higher across the observation period, with less variation across HDs. While our data do not allow us to trace this back to the changes in the legal situation, we observed a considerable jump in coverage in primary care, while other hospital types reported stable coverage. The law mandates employers to only appoint vaccinated staff for caring for vulnerable patients but allows for exemptions and respect of privacy through various occupational health mechanisms. This can be seen as a compromise to navigate the moral dilemma of pitting the interest and rights of the individual HCW against to interests and rights of patients. While the currently high vaccination coverage is promising – also in light of vaccination against coronavirus disease (COVID-19) – it is important to note that the data we analysed only cover acute care hospitals, not long-term care facilities where we strongly suspect vaccination coverage might be lower. In addition, further efforts should be taken to increase the vaccination coverage in secondary care hospitals to at least a similar level as in primary and tertiary care hospitals.

Finally, beyond the additional routine data collection and including long-term care facilities, it would be beneficial to study how HCWs’ attitudes to influenza vaccination have been influenced by the introduction of the new communicable diseases act and what their knowledge, attitudes and practices are regarding the new legal basis for semi-mandatory influenza vaccination for people in their profession.
